# The transcription factor *Maz* is essential for normal eye development

**DOI:** 10.1242/dmm.044412

**Published:** 2020-08-18

**Authors:** Olga Medina-Martinez, Meade Haller, Jill A. Rosenfeld, Marisol A. O'Neill, Dolores J. Lamb, Milan Jamrich

**Affiliations:** 1 Department of Molecular and Cellular Biology, Baylor College of Medicine, Houston, TX 77030, USA; 2 Center for Reproductive Medicine, Baylor College of Medicine, Houston, TX 77030, USA; 3 Molecular and Human Genetics, Baylor College of Medicine, Houston, TX 77030, USA; 4 Baylor Genetics Laboratories, Houston, TX 77021, USA; 5 James Buchanan Brady Foundation Department of Urology, Weill Cornell Medical College, New York City, NY 10065, USA; 6 Englander Institute for Precision Medicine, Weill Cornell Medical College, New York City, NY 10065, USA; 7 Center for Reproductive Genomics, Weill Cornell Medical College, New York City, NY 10065, USA

**Keywords:** *Maz*, *Sfrp2*, Wnt pathway, Eye, CNV 16p11.2

## Abstract

Wnt/β-catenin signaling has an essential role in eye development. Faulty regulation of this pathway results in ocular malformations, owing to defects in cell-fate determination and differentiation. Herein, we show that disruption of *Maz*, the gene encoding *Myc*-associated zinc-finger transcription factor, produces developmental eye defects in mice and humans. Expression of key genes involved in the Wnt cascade, *Sfrp2*, *Wnt2b* and *Fzd4*, was significantly increased in mice with targeted inactivation of *Maz*, resulting in abnormal peripheral eye formation with reduced proliferation of the progenitor cells in the region. Paradoxically, the Wnt reporter TCF-Lef1 displayed a significant downregulation in *Maz*-deficient eyes. Molecular analysis indicates that *Maz* is necessary for the activation of the Wnt/β-catenin pathway and participates in the network controlling ciliary margin patterning. Copy-number variations and single-nucleotide variants of *MAZ* were identified in humans that result in abnormal ocular development. The data support *MAZ* as a key contributor to the eye comorbidities associated with chromosome 16p11.2 copy-number variants and as a transcriptional regulator of ocular development.

## Abstract (precis)

**Summary:** Our study has uncovered *Maz* as an important regulator of eye development in humans and mice, striving to elucidate the role of this gene in eye abnormalities associated with the human ch16p11.2 microdeletions and microduplications.

## INTRODUCTION

Formation of the eye is an evolutionarily conserved developmental process that leads to morphogenesis of the retina, lens and associated eye structures. Disturbances within the eye's developmental cascade are responsible for a spectrum of ocular abnormalities, often leading to partial or complete loss of vision. The Wnt pathway is an established, crucial and conserved signaling pathway in eye morphogenesis. Wnt pathway signaling is essential for multiple developmental events during embryogenesis, regulating cell proliferation and fate decisions (Liu et al., 2007; Livesey and Cepko, 2001). The pathway also has a fundamental role in human diseases (Wang et al., 2019). Wnt proteins activate target cells by binding to the Frizzled (Fzd)/low-density lipoprotein receptor related protein (LRP) complex at the surface of the cell (MacDonald et al., 2009; Nusse and Varmus, 1992). The activated Fzd/LRP complex transduces Wnt signaling into the cell through canonical and non-canonical signaling pathways. The canonical Wnt/β-catenin pathway acts through β-catenin as a transcriptional cofactor, whereas the non-canonical pathway [Wnt/planar cell polarity (PCP) and Wnt/Ca^2+^ pathway] through FZD receptors and/or ROR1/ROR2/RYK co-receptors activates the PCP, receptor tyrosine kinase (RTK) or Ca^2+^ signaling cascades (Katoh, 2017).

Antagonistic regulation of extracellular Wnt signaling is achieved through two broad classes of secreted proteins: one of these is collectively referred to as Dickkopf (Dkk) and the other is the secreted frizzled-related protein (Sfrp) class (Kawano and Kypta, 2003). Members of the Dkk class inhibit the binding of Wnt to the co-receptors LRP5/6 to antagonize Wnt signaling. Members of the Sfrp class can bind directly to Wnt ligands through an extracellular cysteine-rich domain with homology to the Fzd receptors, and thus alter their ability to bind and activate Fzd.

In the developing eye, Wnt signaling controls multiple developmental and morphogenic patterning processes. These processes include the dorsoventral patterning of the optic cup, the lens, the retinal pigment epithelium (RPE), the vascular system and the ciliary margin (CM) (Carpenter et al., 2015; Drenser, 2016; Fuhrmann, 2008; Fujimura, 2016; Hagglund et al., 2013; Liu et al., 2007; Wang et al., 2019). The peripheral rim of the optic cup is the point where the non-pigmented inner layer and the pigmented outer layer meet, and is a unique region of the eye cup that forms two peripheral tissues, specifically the ciliary body (CB) and the iris (Davis-Silberman and Ashery-Padan, 2008). Gain- and loss-of-function experiments, both *in vitro* and *in vivo*, demonstrate that Wnt/β-catenin signaling is required for the proper formation of these structures and for the proliferation of progenitors in the CM (Cho and Cepko, 2006; Liu et al., 2003, 2007). Wnt2b, expressed in the dorsal RPE, and Fzd4 are both bona fide candidates that regulate CB and iris formation, as well as control the expansion of stem cells (Kubo et al., 2003, 2005). Wnt/β-catenin activity is tightly controlled in the peripheral retina by Foxg1, Sox2, Axin2 and Sfrp1/2 (Alldredge and Fuhrmann, 2016; Esteve et al., 2011; Fotaki et al., 2013; Heavner et al., 2014; Matsushima et al., 2011). Although Sfrps antagonize Wnt signaling by sequestering Wnt proteins, they might have alternative roles as the Sfrp1/2 compound null mutant showed inactive Wnt signaling and phenotypic alteration of the peripheral retina displaying abnormal neural retina characteristics (Esteve et al., 2011).

*Maz*, a gene encoding Myc-associated zinc-finger transcription factor, is expressed ubiquitously during development and is required for normal genitourinary (GU) development (Haller et al., 2018). The MAZ protein binds to purine-rich promoters that contain a consensus sequence similar to the one found in promoters regulated by Wilms’ tumor 1 (WT1) protein. WT1 modulates mitogen-activated protein kinase (MAPK) signaling and the Wnt pathway (Kim et al., 2009). *In vitro* knockdown of *MAZ* in HEK293 cells results in differential expression of several WNT morphogens required for normal GU development (Haller et al., 2018). Much less is known about the role of *Maz* in the developing eye. Here, we show that reduction or elimination of *Maz* expression in mice leads to a spectrum of eye phenotypes. We provide evidence that *Maz* exerts its role modulating Wnt pathway activity during eye development by suppressing the expression of Sfrp2 in the developing CM. Using a combined approach of exome sequencing (ES) and copy-number variant (CNV) analysis, we identified mutations in the human homolog *MAZ* underlying ocular disorders. Our results reveal that *Maz* is an important component of the molecular pathways controlling eye development.

## RESULTS

### *Maz* is expressed in the developing mouse eye

*In situ* hybridization (ISH) of *Maz* at different stages of mouse development revealed ubiquitous expression in developing tissues, including the eye (Haller et al., 2018). At embryonic day (E)10.5, *Maz* is found in the embryonic eye with higher levels in the dorsal retina. At E14.5, *Maz* expression is in the neuroretina and in the CM, whereas in the lens, expression is restricted to the anterior lens epithelium (Fig. S1). At later stages [E16.5 to postnatal day (P)10], *Maz* transcripts are found in the outer neuroblastic layer with some scattered cells showing higher expression, whereas in the adult eye, *Maz* was detected in Müller glia cells (Blackshaw et al., 2004).

### *Maz* is essential for proper eye development

**Fig. 1.**
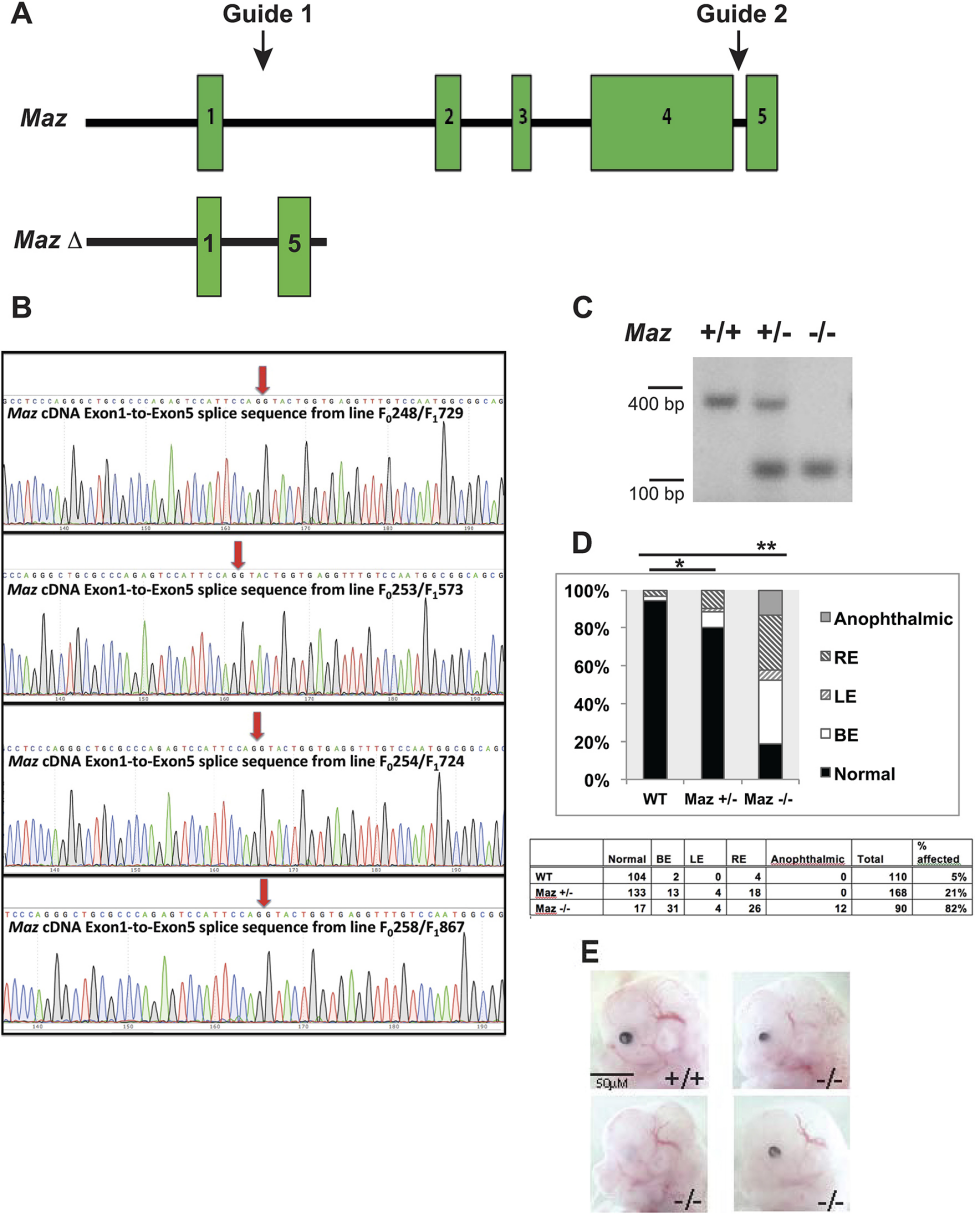
**Strategy of CRISPR/Cas9-based mutagenesis of *Maz* and the resulting mutant eye phenotypes.** (A) Overview of the *Maz* gene and changes generated in knockout mutants. Guide RNAs were designed flanking exons 2 to 4, which encode all of the C2H2 zinc-finger DNA-binding regions of the *Maz* protein. (B) Four independent mutant alleles were obtained and subjected to sequencing. Although they showed slightly different DNA sequences, all four mutant alleles generate identical mRNA with *Maz* exon 1 spliced to exon 5. This splicing event produced a premature stop codon in exon 5. (C) PCR analysis of the *Maz* allele after germ-line transmission. Tail genomic DNA was analyzed by PCR and the corresponding WT and mutant bands were obtained. (D) Association of ocular phenotypes with *Maz* mutant genotypes in C57BL/6J background. Eye phenotypes were observed in 5% of WT mice, 21% of *Maz* heterozygous mice and in 82% of *Maz*-deficient mutants. The phenotype might be present in the right eye (RE), left eye (LE) or in both eyes (BE). Bars show a significant difference: *P=0.015 for *Maz* heterozygotes and **P<0.00001 for *Maz*-deficient embryos. (E) Embryos (E12.5) lacking *Maz* show microphthalmia, anophthalmia and coloboma. The phenotypes are similar in penetrance and expressivity in the four independent *Maz*-deficient lines (see Table S2).

**Fig. 2.**
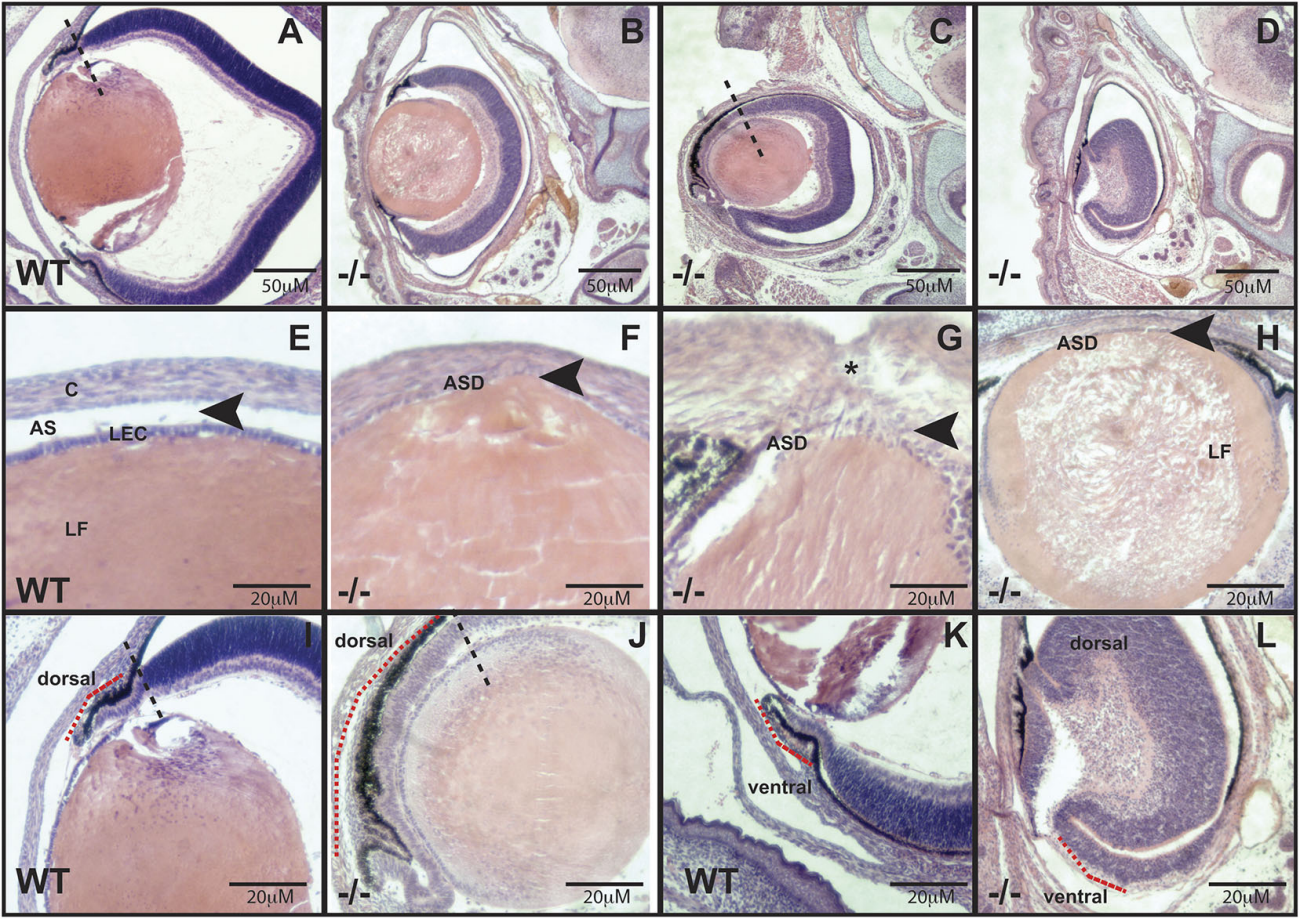
**Loss of *Maz* function causes variable eye defects including microphthalmia, anophthalmia and coloboma.** (A-L) Coronal Hematoxylin and Eosin (H&E)-stained sections through eyes of E18.5 WT (A,E,I,K) or *Maz*^−/−^ embryonic microphthalmic eyes (*n*=8-10 eyes per genotype) (B-D F-H,J,L) showing a variety of abnormalities. Different degrees of microphthalmia are shown in B (less affected) to D (more affected) compared with the WT eye in A. Mutants show anterior segment dysgenesis. Arrowheads indicate the presence of the anterior chamber in the WT eye (E) and its absence in *Maz* mutants (F-H). In addition, a persistent keratolenticular connection between the lens epithelium and cornea is present in the mutant lens (black asterisk in G). In several cases, the central lens fiber cells are disorganized (H). Some mutants show ectopic expansion of the peripheral optic cup (red dotted line in J) compared with the thin single-layered morphology in the WT (red dotted line in I). The ventral peripheral cup loses the pigmented RPE morphology observed in the WT optic cup (red dotted line in K) and resembles the peripheral neural retina (L). In this case, both the dorsal and ventral tips of the eye cup show absence of pigment and are thicker than the WT RPE. In these severely affected eyes, there is a complete absence of lens (aphakia) (L). AS, anterior segment; ASD, anterior segment dysgenesis; C, cornea; LEC, lens epithelial cells; LF, lens fibers.

To investigate whether *Maz* deletion results in eye abnormalities, mice harboring a recently described targeted deletion of *Maz* were examined (Haller et al., 2018) (Fig. 1A-C). Mice heterozygous for the deletion (*Maz*^+/−^) were relatively normal, with a slight deviation in survival from Mendelian expectations. However, ∼90% of homozygous null mutants (*Maz*^−/−^) died before weaning, demonstrating that *Maz* activity was essential for postnatal viability. Analysis of *Maz*-deficient embryos at different stages of development showed them to die at the perinatal stage with developmental eye defects. Homozygous inactivation of *Maz* caused a variable eye phenotype in 80% of the mutants, ranging from unilateral microphthalmia, sometimes with coloboma, to bilateral anophthalmia (Fig. 1D,E). Some C57 BL/6J wild-type (WT) animals (5%) exhibit ocular abnormalities, which appear phenotypically different from the previously identified eye phenotype associated with the C57BL/6N genetic background (Mattapallil et al., 2012); however, the penetrance of these abnormalities is significantly increased in a gene-dose-dependent fashion in *Maz^+/−^* (21%) and *Maz^−/−^* (82%) mice (Table S1 and Fig. 1D). Histological analysis of E18.5 *Maz^−/−^* eyes, the latest stage at which *Maz*-deficient embryos can be consistently recovered, revealed a spectrum of mutant eye phenotypes ranging from grossly normal eyes of slightly smaller size to mutants with severely affected eyes with significantly reduced size (Fig. 2B-D) in comparison to the WT eye (Fig. 2A). Mildly affected mutant eyes (Fig. 2D) displayed anterior segment dysgenesis (ASD), abnormal keratolenticular connections with absence of anterior segment (Peter's anomaly) and occasionally abnormal lens fibers. In contrast, the WT eye (Fig. 2E) exhibited the lens and cornea separated by the anterior segment, and the lens presenting both the epithelial cells and organized fibers. In severely affected mutants (Fig. 2D), the microphthalmic eyes were accompanied by different anomalies including ventral coloboma. In some cases, an ectopic expansion of the peripheral dorsal CM was observed (Fig. 2C,J) compared to the dorsal CM in the WT eye (Fig. 2A,I). In extreme cases, both the dorsal and ventral pigmented epithelium were thickened to resemble a mirror image of the subjacent neuroretina in comparison to the normal dorsal (Fig. 2I) and ventral (Fig. 2K) pigmented epithelium in the WT, and the lens was entirely absent (Fig. 2L). In a few extreme cases, the ocular tissue was absent (data not shown).

Phenotypic analysis showed that ∼30% of *Maz^+/−^* adult mice present eye abnormalities with lens opacities observed consistently. Detailed histological comparison of eyes in WT and *Maz^+/−^* adult mice showed that haploinsufficiency of *Maz* causes a significant decrease in retinal thickness and a change in the open angle of the ciliary body (Fig. S2). Homozygous adult mice that survived for a few weeks included two males with no obvious eye phenotype and two runted females with very microphthalmic eyes. Eye tissue was recovered from only one of the affected females and consisted only of RPE (data not shown).

**Fig. 3.**
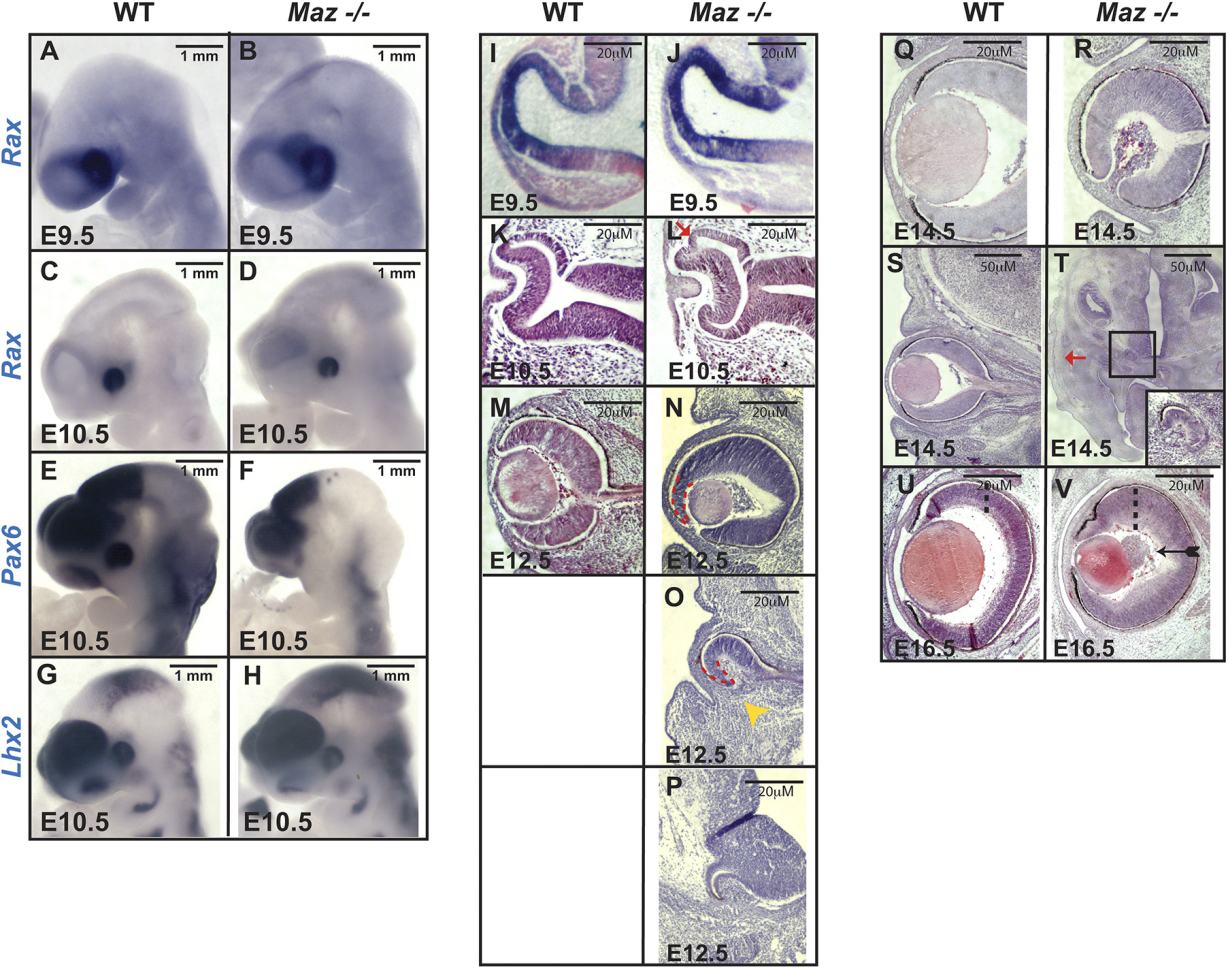
**Histological analysis of eye development in *Maz* mutants.** (A-H) Whole-mount ISH analysis (*n*=6-8 eyes per gene/age analyzed) of *Rax* to WT and *Maz*-deficient embryos at E9.5 (A,B) and E10.5 (C,D) and of *Pax6* (E,F) and *Lhx2* (G,H) to WT and *Maz*-deficient embryos at E10.5. Initially, *Maz* mutants have a relatively normal eye specification. There is no difference between the WT (A) and *Maz* mutant (B) *Rax* at E9.5 and *Pax6* E9.5 expression (data not shown). *Rax* continues with normal expression at E10.5 (C,D), although in some cases (as exemplified by *Pax6* expression at E10.5) the eye field is very small and stays abnormally close to the brain (E,F). *Lhx2* expression at E10.5 also looks relatively normal (G,H). (I,J) ISH analyses of *Pax6* expression on coronal sections of E9.5 show a relatively normal optic vesicle both in WT (I) and *Maz* mutant embryos (J). (K-V) H&E-stained coronal section (*n*=6-8 eyes per embryonic age) at E10.5 (K,L), E12.5 (M-P), E14.5 (Q-T) and E16.5 (U,V). At E10.5, the first stage where there are differences between the WT and *Maz* mutants, the dorsal eye cup hinge region looks overgrown in the mutant (red arrow) when compared with the WT cup. At E12.5, there is clear variable expressivity of the phenotype in the *Maz* mutant eye, ranging from a smaller eye with ectopic expansion of the peripheral optic cup (red dashed lines) and the lens abnormally rotated (N) or coloboma in the ventral eye (yellow arrowhead) with absent lens (O) to embryos apparently anophthalmic, but with a small cup with RPE tissue attached to the brain (P). At E14.5, we observed a similar range of severity of ocular phenotypes with some *Maz* mutant embryos showing the dorsal eye region overgrown with no sign of lens formation (Q,R). In rare cases, some embryos present a micro eye with RPE found abnormally located at the base of the brain (black box) (T) instead of the normal eye in the surface (red arrow) connected to the brain by the optic nerve (S). At E16.5, in *Maz* mutants with mild ocular defects, the central neural retina in the *Maz* mutant (V) is thicker than in the control retina (U) (dashed lines for comparison). In addition, in the mutant there is an abnormal accumulation of angioblasts that normally form the vasculature of the embryonic eye (black arrow in V).

To determine the onset of these abnormalities in eye development, we investigated eye-specific gene expression and morphogenesis during early eye development of *Maz* mutants. During development, the eye field at the anterior neural plate gives rise to the optic vesicles, which then turn into the optic cups. The eye field expresses several genes that are crucial for eye formation, including *Rax*, *Pax6*, *Six3*, *Lhx2*, *Otx2* and *Six6* (Mathers and Jamrich, 2000). When we examined morphogenesis and expression of *Rax*, *Pax6* and *Lhx2* at E9.5 and E10.5, we found that at E9.5 their expression domains were similar in *Maz^−/−^* and WT embryos (Fig. 3A,B,I,J). At this stage of embryonic development, there were no obvious genetic or morphological differences between the developing WT and mutant eyes, as exemplified by *Rax* (Fig. 3A,B) and *Pax6* (Fig. 3I,J) expression.

At E10.5, expression of these genes was still very similar (Fig. 3C-H), although in some cases, as shown by *Pax6* expression, the eye field was very small and located abnormally close to the brain (Fig. 3E,F). At this stage, the optic cups are formed on schedule in both WT and mutant mice, but the first morphological differences between *Maz^−/−^* and WT embryos appear at the periphery of the optic cup. In the *Maz* mutant, the dorsal fold-back hinge-point is overgrown and displaced to the outside of the optic cup (Fig. 3K,L). Beginning at E12.5, major differences appear between WT (Fig. 3Q,S,U) and mutant (Fig. 3R,T,V) eyes. The mutant eyes are smaller and grossly altered with defects in both the dorsal and ventral optic cup (Fig. 3N-P). The expressivity of the phenotype in the mutant is variable, ranging from a smaller eye with ectopic expansion of the peripheral optic cup and the lens abnormally rotated (Fig. 3N) to the complete absence of the ventral region of the eye (Fig. 3O). In a few cases, embryos appear anophthalmic, but sections reveal that they have a small optic cup with RPE tissue attached to the brain (Fig. 3P). By E14.5, the dorsal retina of *Maz* mutant eyes is frequently overgrown and the lenses are either abnormally positioned or absent (Fig. 3Q,R). In some mutant embryos, a rudimentary eye structure can be found close to the base of the brain (Fig. 3S,T). At E16.5, in some mutants the retina is abnormally thick and defects were observed in the extraretinal hyaloid vasculature (Fig. 3U,V).

### *Maz* is required for correct morphogenesis of the ciliary body

To better understand the functional consequences of the inactivation of *Maz* in eye formation, we investigated if the morphology and proliferation of the CM was altered. We queried whether this region had lost its defining characteristics. Gene expression and proliferation in the CM of *Maz* mutants was compared with that observed in WT eyes.

**Fig. 4.**
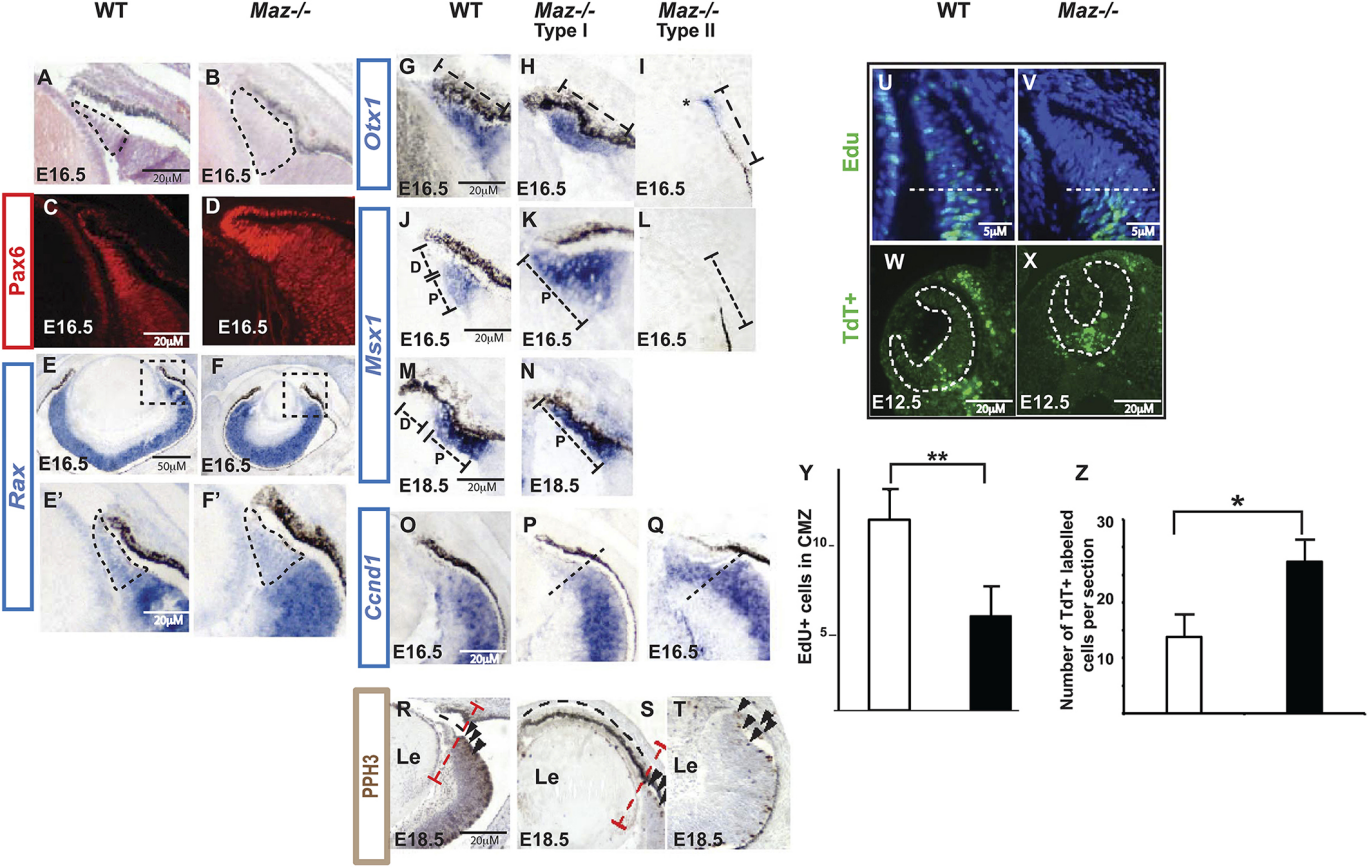
**CM patterning is affected in *Maz* mutants.** (A,B) H&E-stained coronal sections (*n*=6 eyes per genotype) the CM in control (A) and *Maz* mutants (B) shows an abnormal shape of the dorsal CM in *Maz* mutants. (C,D) Pax6 immunostaining (*n*=6 eyes per genotype) showing the high expression domain in the dorsal CM of E16.5 WT (C), whereas the mutant embryo shows an abnormal morphology in the high Pax6 expression domain (D). (E-F′) ISH of Rax (*n*=6 eyes per genotype) shows high expression in the neuroretina and lower expression in the CM of WT embryos (E,E′) and an enlarged low expression domain in the *Maz* mutant (F,F′). The box delineates the margin of the optic cup shown in E′ and F′. (G-I) ISH analysis of coronal sections (*n*=8 eyes per genotype) demonstrates that *Otx1* is expressed in the entire CM in both control (G) and in *Maz* mutant type I (H). However, the *Otx1* expression domain in the most hypoplastic mutant retinas (2 of 8) is markedly decreased in *Maz* mutant type II (marked by an asterisk) (I). (J-N) In WT embryos, *Msx1* expression is restricted to the most proximal ‘P’ region of the CM, but is absent in the distal ‘D’ region in E16.5 (J) and E18.5 (M) peripheral retinas. In the CM of *Maz* mutants, *Msx1* expression is expanded to the distal domain (K,N) and is absent in the most hypoplastic retinas (2 of 8) (L). (O-Q) In the WT retina and in *Maz* mutant type I, *Ccnd1* expression is strongest in the neural retina with lower or absent expression in the CM. The dashed line delimits the region of strong *Ccnd1* expression (O,P). Transition of *Ccnd1* from high to low expression is absent in the most hypoplastic *Maz*-deficient retinas. Instead, CM displays high expression of *Ccnd1*, which is normally present in the neural retina (Q). (R-T) PPH3 immunostaining in retinal sections of E18.5 embryos (*n*=8 eyes per genotype). In the CM of control embryos (R), there are few or no PPH3-positive cells and the CM is defined by its thinner morphology (black dashed line). CM is clearly delimited (red dashed line indicates the boundary) from the neuroretina, which contains a high number of PPH3-positive cells (black arrowheads). In some *Maz* mutants, the CM is abnormally expanded (S), whereas in other mutants the boundary between the CM and neuroretina is absent and the periphery of the retina displays characteristics of a neural retina, including morphology, hypopigmentation and presence of PPH3 cells (T). (U-X) Proliferation is significantly reduced in the CM of *Maz*^−/−^ in comparison with the control counterparts (*n*=6 eyes with three technical replicates). Representative section of peripheral WT (U) and *Maz*^−/−^ (V) retinal sections of mice injected with EdU at E14.5 show significantly reduced Edu incorporation in the CM of *Maz* mutants. Increased cell death was observed in the optic cup of E10.5 *Maz*^−/−^ (X) compared with the WT littermates (W). (Y) The number of EdU-positive cells in the CM of WT and *Maz* mutants is given as the average cell count per CM (*n*=6-8 eyes with three technical replicates per genotype). Error bars represent mean±s.e.m. Student's two-tailed unpaired *t*-test: **P<0.05. (Z) The number of TUNEL/fluorescein-labeled apoptotic cells in WT and *Maz*^−/−^ E10.5 eyes (*n*=6 eyes per genotype with three technical replicates per genotype) is shown as the average TdT-positive cells per section±s.e.m. (*P<0.05 Mann–Whitney *U-*test).

The peripheral CM, from which the ciliary body and iris are derived, is characterized by a high level of *Pax6* expression (Matsushima et al., 2011), expression of *Otx1* (Martinez-Morales et al., 2001) and *Msx1* (Monaghan et al., 1991), as well as an absence of expression of the cell-cycle regulator *Ccnd1*. This region also displays a low proliferation rate, as diagnosed by low 5-bromo-2'-deoxyuridine (BrdU) incorporation (Trimarchi et al., 2009) and by the relative absence of phosphohistone H3 (PPH3)-positive cells. At E16.5, *Pax6* expression is highest in the distal tips of the retina of both the WT and *Maz* mutant eyes (Fig. 4C,D). However, *Pax6-*expressing cells are irregularly positioned in the CM of *Maz* mutants with an abnormal thickness (Fig. 4D) compared to the WT retina (Fig. 4C). This abnormal morphology is also observed in H&E-stained CM coronal sections (Fig. 4A,B). Expression of the retinal progenitor cell marker *Rax* revealed that this gene is normally expressed in the prospective neuroretina with lower levels in the CM (Fig. 4E,F), but the area of low *Rax* expression is enlarged in mutant embryos (Fig. 4E′,F′).

Although there is a wide range of ocular defects in *Maz* mutants, comparison of expression of specific markers of the CM showed that molecularly there are two different phenotypes. In both WT and in mildly affected embryos (type I), *Otx1* is expressed in the entire CM (Fig. 4G,H). In contrast, in the most hypoplastic *Maz^−/−^* eyes (type II), there is a clear reduction of *Otx1* expression in the CM (Fig. 4I). *Msx1*, a gene that defines the posterior domain of the CM (Belanger et al., 2017; Marcucci et al., 2016), is expressed in the proximal region of the CM but is absent in the distal region of the peripheral retinas in WT embryos at E16.5 (Fig. 4J) and E18.5 (Fig. 4M). In the CM of *Maz* mutants, *Msx1* expression is expanded to the distal domain of mildly affected mutants (Fig. 4K,N) and is absent in the most hypoplastic retinas (Fig. 4L). In the WT retina (Fig. 4O) and in the type I phenotype (Fig. 4P), *Ccnd1* expression is strongest in the neural retina and absent in the CM. In the mutant type II embryos, the CM displays an abnormally high expression of *Ccnd1* (Fig. 4Q).

PPH3 immunostaining in retinal sections of WT E18.5 embryos showed few or no proliferating PPH3-positive cells in the thinner CM, although there were many PPH3-positive cells in the neuroretina (Fig. 4R). In *Maz* mutants, no PPH3-positive cells were observed in the long ectopic expansion of the peripheral cup (Fig. 4S), whereas in severely affected mutants the characteristic thin region of the CM was absent and the periphery displayed the characteristic PPH3-positive cells of the neural retina (Fig. 4T).

We also evaluated the incorporation of 5-ethynyl-2′-deoxyuridine (EdU) in the CM of *Maz*-deficient retinas. We observed a significant decrease in the number of EdU-positive cells in the ciliary marginal zone (CMZ) of *Maz*^−/−^ retinas (Fig. 4U,V,Y). One explanation for the loss of EdU-positive cells in the CM might be selective cell death. To determine if mutant CM cells exhibit increased apoptosis, we performed TUNEL reaction on sectioned WT and mutant eyes. No accumulation of apoptotic TUNEL-positive cells was observed in the CM of E14.5 (data not shown) or E12.5 embryos. In contrast, there was a significant increase in TUNEL-positive cells observed in the ventral region of the *Maz*-deficient eye cups compared with those in the control (Fig. 4W,X,Z).

These results show that in the absence of *Maz* activity the patterning of the CM is affected. These findings also raised the possibility that during eye development Maz is regulating Wnt signaling, as both the *Msx* and *Otx1* genes are known targets of this cascade (Liu et al., 2007; Willert et al., 2002).

### Maz regulates canonical Wnt signaling in the retina

**Fig. 5.**
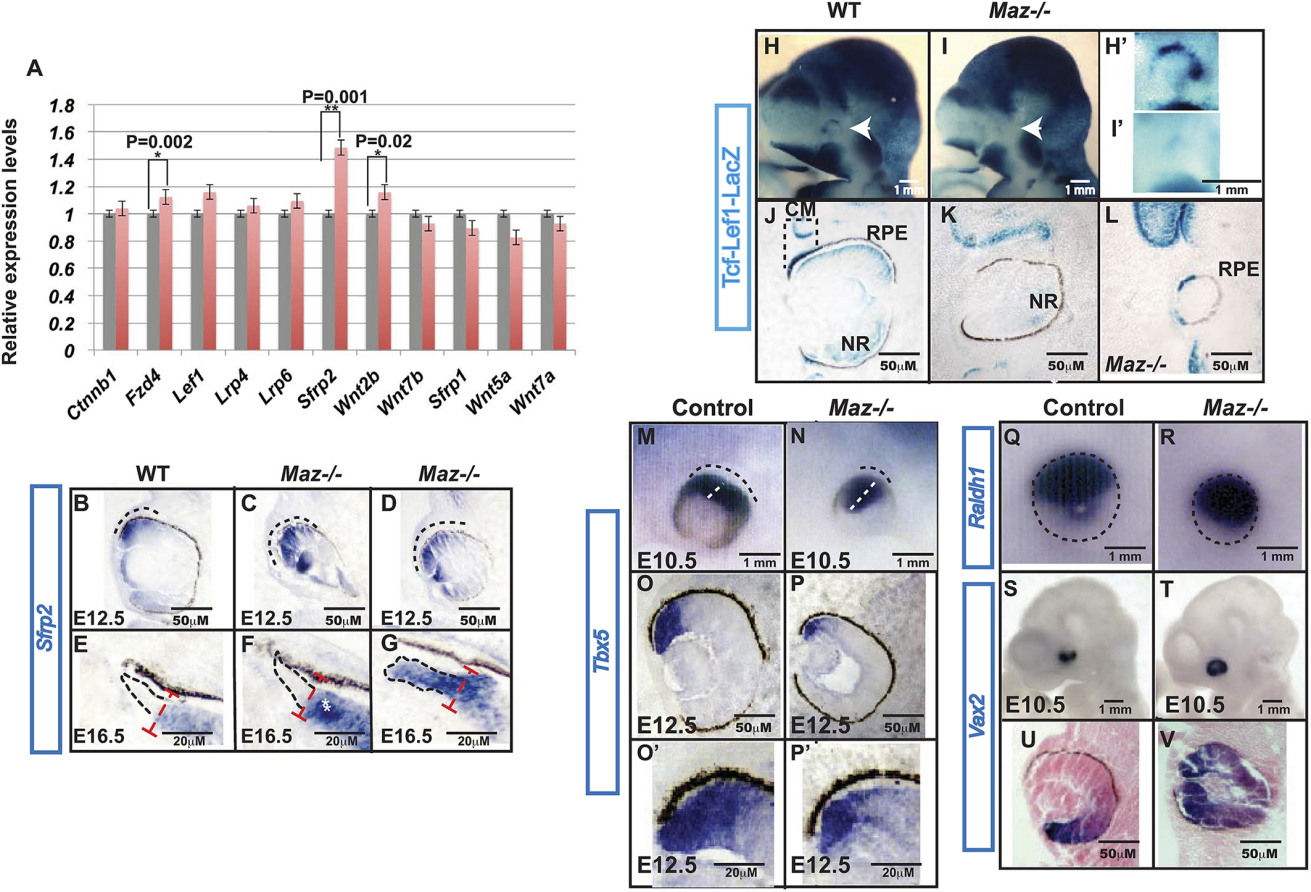
**Changes in *Wnt* expression and *Wnt* reporter activity in the *Maz* mutant.** (A) Quantitative comparison of expression levels by qPCR of several *Wnt* genes or *Wnt* modulators expressed in the eye. Analysis shows a significantly higher expression of *Sfrp2*, *Fzd4* and *Wnt2b* in the eye of *Maz* mutants (pink bars) compared with the WT (gray bars). (B-G) Three biological replicates in triplicate normalized to GAPDH as indicated. ISH for the *Sfrp2* gene demonstrates an expansion of the high expression zone in the peripheral retina of *Maz* mutants (C,D) when compared with the control at E12.5 (*n*=6 eyes per genotype/age) (B). In *Maz*-deficient embryos at E16.5, *Sfrp2* is upregulated in the zone adjacent to the CM (asterisks indicate the Sfrp2 high-level expression region) (F). This upregulation is absent in the CM of control retinas (E). In some mutants, *Sfrp2* expression reached the CM zone where it is normally absent (G). (H-L) Downregulation of dorsal Wnt/β-catenin signaling (white arrowheads) was observed in the developing eye of E10.5 TCF-Lef1-LacZ:Maz^−/−^ (*n*=6 eyes per genotype) compound mutants (I,I′) compared with the WT embryos (H,H′). Coronal sections of E12.5 (*n*=13-14 eyes per genotype) were stained with X-gal to detect LacZ activity. In the control eye (TCF-Lef1-LacZ:Maz^+/+^), activity of LacZ was detected in the prospective CM, central neuroretina (NR) and the retinal pigment epithelium (RPE) (J). In contrast, in the TCF-Lef1-LacZ:Maz^−/−^ embryos, ^ ^LacZ activity was lost in the entire eye of some mutants (K). In others, it persisted in the RPE only (2 of 13) (L). (M-V) Whole-mount ISH analysis (*n*=6-8 eyes) of *Tbx5* (M,N), *Raldh1* (Q,R) and *Vax2* (S,T). *Tbx5* (O,P and O′,P′) and *Vax2* (U,V) ISH analysis on coronal sections of E12.5 optic cups from control and *Maz* mutant, respectively.

Canonical Wnt signaling (Wnt/β-catenin) is essential for eye development. It is not only required for the transition of the optic vesicle to the optic cup, but also to promote the differentiation of the RPE and to maintain dorsal retinal identity (Fujimura, 2016; Hagglund et al., 2013). Additionally, canonical Wnt signaling is implicated in the development of the CM in several species, including mice and chickens (Cho and Cepko, 2006; Liu et al., 2003, 2007). Altered Wnt/β-catenin signaling results in numerous ocular malformations (Fuhrmann, 2008; Fujimura, 2016). Several Wnt signaling family members are active in the developing peripheral retina, such as Wnt2b, Fzd4 and Lef1 (Liu et al., 2003). Maz is a transcription factor that binds G-rich consensus sequences similar to those bound by WT1, a known Wnt pathway regulator (Kim et al., 2009). On the basis of this similarity, we hypothesized that Maz regulates Wnt pathway genes that are required for the correct patterning of the eye. Wnt/β-catenin activity is present in the neuroblastic layer, the RPE, the distal portion of the optic cup and the peripheral retina, where it is controlled by Sfrp1 and Sfrp2 (Esteve et al., 2011). To evaluate how the Wnt pathway is affected in the embryonic eyes of *Maz* mutants, a quantitative real-time PCR (qPCR) analysis was performed in *Maz* mutant and WT eyes using specific primers for several members of the *Wnt* cascade and its regulators, *Sfrp1/2* (Cho and Cepko, 2006; Liu et al., 2003). Three genes, *Wnt2b*, *Fzd4* and *Sfrp2*, were significantly upregulated in *Maz* mutants (Fig. 5A). Wnt2b, possibly using Fzd4 as a receptor, functions to establish the formation of the CM and also to keep a pool of CM progenitor cells in an undifferentiated state (Kubo et al., 2003, 2005). Sfrp2 belongs to a type of secreted regulatory proteins for Wnt signaling and has homology with the Wnt receptor Frizzled; Sfrp2 is generally accepted as an antagonist that binds and sequesters Wnt ligands to prevent signal activation. However, Sfrp1 and Sfrp2 are also required for the activation of Wnt/β-catenin signaling in the peripheral retina (Esteve et al., 2011). Therefore, our results suggest that Maz is a regulator of the Wnt pathway through the regulation of Wnt signaling members, including the modulator Sfrp2.

To confirm that *Sfrp2* expression is increased in the *Maz* mutants, *Sfrp2* expression was monitored by ISH (Fig. 5B-G). At E12.5, the *Sfrp2* expression domain was predominantly located in the dorsal retina of WT embryos (Fig. 5B) and expanded in the *Maz* mutants (Fig. 5C,D). In E16.5 WT embryos, *Sfrp2* expression is confined to the central retina. Although there is a gradient of expression, with higher expression close to the CM, the CM itself does not express the *Sfrp2* gene (Fig. 5E). In some of the *Maz* mutants *Sfrp2* expression is relatively normal compared with that in WT (Fig. 5F), but in some embryos *Sfrp2* expression is expanded into the CM (Fig. 5G). These findings suggest that Maz possibly acts through inhibition of *Sfrp2* expression in the CM.

To test the hypothesis that Maz normally suppresses *Sfrp2* to promote Wnt activity in the CM, compound embryos with T-cell factor/lymphoid enhancer factor-β-galactosidase (TCF-Lef1-LacZ), a canonical Wnt/β-catenin reporter, were analyzed. These mice carry LacZ under the control of a minimal heat shock protein (HSP) promoter and six copies of the TCF-Lef1 responsive element (Liu et al., 2003). E10.5-E12.5 *Maz*^−/−^/TCF-Lef1-LacZ+ and control littermates *Maz*^+/+^/TCF-Lef1-LacZ+ were evaluated for LacZ activity. In control embryos at E10.5, TCF-Lef1 activation was detectable in the dorsal optic vesicle (Fig. 5H,H′), whereas at E12.5 activation was found in the neuroblast layer of the neuroretina, the CM and the RPE (Fig. 5J). In contrast, in E10.5 *Maz* mutant eyes (*n*=6), LacZ was almost absent in the dorsal part of the optic vesicle (Fig. 5I,I′), whereas in E12.5 *Maz* mutants (11 of 13) Wnt reporter activity was reduced in the whole retina, including the CM and RPE (Fig. 5K). In a few cases (2 of 13), Wnt activity in the eye was observed only in the RPE (Fig. 5L).

To further confirm loss of Wnt/β-catenin activity in the embryonic eye of *Maz* mutant eyes, we evaluated if the dorsoventral patterning of the retina was affected. *Tbx5* (Fig. 5M) and *Raldh1* (Fig. 5Q) are normally expressed in the dorsal domain of the optic vesicle at E10.5. The expression of both genes is preserved in *Maz* mutants, but while the expression of Raldh1 is relatively normal, the expression domain of *Tbx5* appears thinner and elongated (Fig. 5N,R). At E12.5, the expression domain of *Tbx5* is significantly reduced and thinner in comparison to the control embryos (Fig. 5O,P). This observation suggests that the induction of dorsal identity is not affected, but the activity of *Maz* is important for its maintenance. Furthermore, it was observed that expression of the ventral-specific gene *Vax2* is extended into the dorsal domain, where normally it is absent (Fig. 5S-V). At E10.5, essentially all the cells in the mutant neuroretina adopt a ventral identity (Fig. 5V). These results suggest that in the *Maz* mutants there is a partial ventralization of the retina, as expression of the dorsal genes *Tbx5* and *Raldh1* is preserved. Therefore, the diminished Wnt/β-catenin reporter activity, together with the partial loss of dorsal identity in the retina of *Maz* mutants, supports the conclusion that Maz is a regulator of Wnt signaling and is essential for the robust formation of the D-V patterning and the correct specification of the CM. Our findings suggest that this regulation, at least in the peripheral retina, is primarily through suppression of *Sfrp2*.

### Human *MAZ* is a candidate gene in the 16p11.2 chromosomal region for ophthalmic comorbidities in humans

In humans, ocular diseases have not been directly associated with *MAZ* mutations. Screening CNVs in patients with autism and cognitive disorders led to identification of the deletions and duplications of chromosome 16p11.2 as one of the most frequent genetic causes for autism spectrum disorders, schizophrenia and other neurodevelopmental disorders (Miller et al., 1993; Weiss et al., 2008). More recently, CNVs in this region were associated with urogenital (Haller et al., 2018) and microphthalmia, anophthalmia and coloboma (MAC) ocular malformations (Bardakjian et al., 2010; Hernando et al., 2002).

To understand the role of *MAZ* in human ocular morbidities, we searched the literature and cases reported in the Database of Chromosomal Imbalance and Phenotype in Humans Using Ensembl Resources (DECIPHER). This database catalogs potentially pathogenic genomic changes in patients. There were 580 patients harboring deletions and duplications in the region encompassing *MAZ*, with only 391 providing data on clinical features. From those cases with available clinical data, 44 patients exhibit various ophthalmic malformations. Excluding patients with refractive errors (hypermetropia, myopia and astigmatism) and/or strabismus, only 22 of 391 (5.6%) CNV carriers (eight duplications; 14 deletions) exhibit ocular disorders (Fig. S3). The frequency of ocular morbidities in this analysis is similar to that previously reported (2.1%) from a cohort of 357 carriers of the 16p11.2 BP4-BP5 deletion and 68 intrafamilial, non-carrier controls (Zufferey et al., 2012).

Owing to the fact that MAZ is a transcription factor that regulates expression of other transcription factors and signaling molecules, *MAZ* is a candidate contributor to ocular malformations associated with 16p11.2 CNVs. Interestingly, knockdown of the 16p11.2 homolog genes *coro1a*, *maz or fam57b* in zebrafish results in small eyes with protruding lenses (Blaker-Lee et al., 2012; Schmitt and Dowling, 1994). Thus, *MAZ* was considered a strong candidate gene for both its location and function.

### ES identified novel *MAZ* variants in individuals with a variety of eye anomalies

**Fig. 6.**
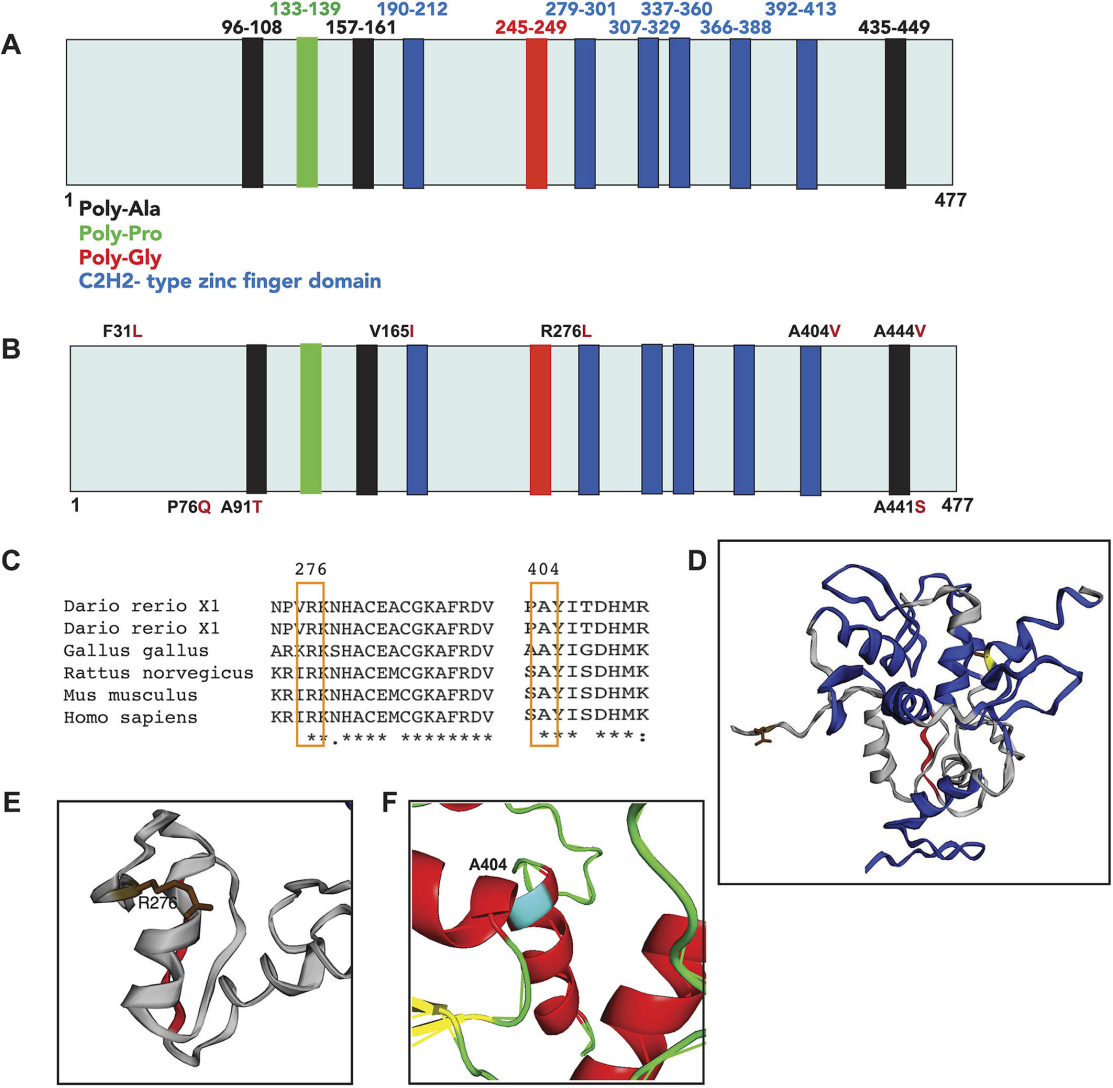
**Functional domains, novel mutations** **and crystal structure of Maz** **protein.** (A) Schematic representation of the functional domains of Maz protein. (B) Novel mutations are shown with the amino acid position in black and the change found in red. (C) Clustal omega alignment of the variants R276L and A404V highlighted in yellow to show complete conservation across different species. (D) Crystal structure modeling of Maz (Phyre2 PDB: c2kmkA). (E) Maz protein containing R276 (brown) in the vicinity of the α-helix of the second zinc-finger and the polyglycine domain. The substitution R276L involves a basic/aliphatic amino acid replacement, predicted to have a deleterious effect on the protein. (F) The amino acid A404 is shown in blue in the last zinc finger of the Maz protein. The conservative A404V sequence change involves exchanging an alanine residue, which is considered one of the best helix-forming residues, for the poor helix-forming residue valine in the zinc-finger domain; therefore, this change is predicted to have a potential functional effect.

We performed a retrospective analysis of ∼11,500 consecutive individuals undergoing clinical ES to determine if *MAZ* variants are associated with eye abnormalities. Importantly, this cohort is enriched for structural congenital defects. A total of 75 rare (with allelic frequency <0.01) or novel [never reported in The Genome Aggregation Database (gnomAD)] *MAZ* variants were identified. Some 38 patients with novel variants (six of them with eye phenotype) were excluded, as they had variants in another gene that probably caused their phenotype. A total of 37 non-synonymous coding single-nucleotide variants (SNVs) were identified and their frequencies were retrieved from gnomAD (Lek et al., 2016). As summarized in Table 1, seven of the 37 individuals with *MAZ* variants (19%) were affected with different eye disorders (including cone-rod dystrophy, rapid progressive vision loss, retinitis pigmentosa, congenital cataracts and optic nerve hypoplasia). The dominant phenotype observed in these *MAZ* variants is consistent with the ocular defects observed in the heterozygous *Maz* loss-of-function mice. We selected three *MAZ* variants predicted to be damaging and/or not tolerated, using the Polyphen2 and SIFT programs for structural analysis. We noted that variant A446V is located in the C-terminal polyalanine domain of the protein (Fig. 6A,B). Several studies have suggested that this domain might have a role in transcriptional repression in proteins like *kruppel* and *engrailed* in *Drosophila* and in the glucocorticoid receptor in humans (Han and Manley, 1993; Lavoie et al., 2003; Licht et al., 1990). Two additional variants, R276L and A404V, affect amino acids highly conserved from zebrafish to humans (Fig. 6C). The three-dimensional structure of the human WT, R276L and A404V variants of *MAZ* were modeled by the comparative protein method (Fig. 6D-F). Both variants are predicted to perturb local interactions and affect the function of the protein. The non-conservative substitution of R276L, involving a basic/aliphatic amino acid replacement, was predicted to have a deleterious effect on the protein structure (Fig. 6E). The semiconservative A404V sequence change involves exchanging an alanine, which is considered one of the best helix-forming residues, for a weaker valine within the zinc-finger domain and is therefore predicted to have a potential functional effect (Fig. 6F). The results suggest that these SNVs represent potentially pathogenic *MAZ* alleles, although functional studies will be necessary to discard the possibility of an occurrence by chance. In support, *MAZ* presented a probability of loss-of-function intolerance (pLI) of 0.94 and an o/e score of 0.07. These scores are indicative of a gene with strong intolerance to loss-of-function, where only 7% of the expected loss-of-function variants were observed. Together, these findings implicate rare and novel variants of *MAZ* as potential causative variants associated with eye abnormalities. Moreover, the functional analysis of the *Maz* mutant mouse model and the evidence that *Maz* regulates the Wnt/β-catenin pathway support the role for *MAZ* variants in human eye disease.

**Table 1.**
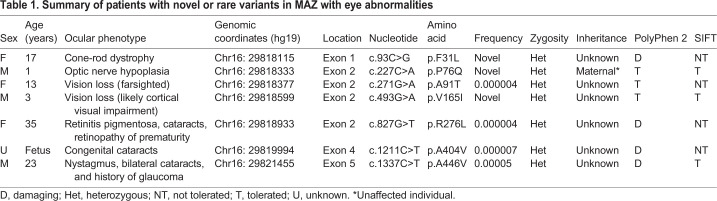
**Summary of patients with novel or rare variants in MAZ with eye abnormalities**

## DISCUSSION

### *Maz* activity is essential for normal eye development

In this study, we have demonstrated that *Maz* function is essential for correct eye patterning during development. This was achieved in both a mouse model with a targeted deletion of *Maz* (Haller et al., 2018) and by genomic studies of human patients. We show that disruption of *Maz* results in microphthalmia, coloboma and a range of eye abnormalities with varying expressivity and incomplete penetrance in mice, and outline a molecular mechanism whereby *Maz* regulates *Wnt* activity in the developing eye. First, *Maz* was found to be necessary for the activation of the Wnt TCF-Lef1 reporter. Second, we demonstrated that genetic deletion of *Maz* leads to defective dorsoventral patterning in the optic vesicle and abnormal differentiation of the CM, which involved the Wnt pathway. Our observations suggest that the canonical Wnt/β-catenin pathway is normally upregulated by *Maz* in the eye. However, disruption of Wnt/β-catenin activity in *Maz* mutants cannot be explained by attenuation of Wnt signaling members, as expression of the key factor establishing the dorsal retina *Lrp6* is not changed and *Wnt2b* and *Fzd4* (the candidates to promote CM formation and maintenance of the progenitors) are significantly upregulated in the CM of *Maz* mutants. Of note, increased *Wnt2/Fzd4* expression was insufficient to activate the Wnt reporter and induce a correct CM in the *Maz* mutant. This might reflect an absent reinforcing synergistic signal or, alternatively, the presence of an inhibitor of the inducing effect of *Wnt2b/Fzd4* overexpression. Our data implicated the latter, as *Sfrp2* overexpression can effectively inhibit Wnt signaling both *in vivo* and *in vitro*, its overexpression in the *Maz* mutant might be sufficient to abrogate Wnt activity. The enhanced expression of *Wnt2b* and *Fzd4* in the mutant indicates that *Maz* function is required for the correct regulation of these genes and might involve a negative feedback, potentially explaining the increased *Wnt2b/Fzd4* expression of these genes in the *Maz* mutants.

### Maz is a regulator of Wnt activity: the role of Sfrp2

Sfrps modulate the Wnt cascade by their interaction with both Wnt ligands and their receptors (Esteve and Bovolenta, 2010). *Sfrp2* is not expressed in the CM, but it is expressed in the undifferentiated retinal neuroepithelium: its expression is progressively downregulated in the central retina forming a central (low) to peripheral (high) gradient. *Sfrp1* is mainly expressed in the RPE and the CM (Marcos et al., 2015). In the CM, Sfrp2 cooperates with Sfrp1 to enhance activity of Wnt/β-catenin signaling, possibly through the promotion of interactions of Wnt ligands with their *Fzd* receptors (Esteve et al., 2011; Lopez-Rios et al., 2008).

Loss of *Maz* function results in an upregulation of *Sfrp2* and a variable CM phenotype. Some mutants showed a reduction of the CM markers, whereas others had an abnormal distoproximal patterning of the CM. This observation indicated that *Maz*, possibly through its influence on *Wnt* signaling, was essential for correct establishment of the neuroretina-CM boundary as well as for patterning of the CM. Several studies indicate that Wnt/β-catenin signaling activity in the peripheral retina is controlled by Sfrp1/Sfrp2 to establish the border between the peripheral and central neural retina. Eyes of *Sfrp1^−/−^ Sfrp2^−/−^* compound mutants lack the retinal periphery domain and show an increased number of retinal ganglion cells (Esteve et al., 2011).

Based on the proposed role of Sfrps in the CM and increased expression of Sfrp2 in the *Maz* mutants, we expected Sfrp2 expression to upregulate the TCF-Lef1-LacZ reporter in the CM. Paradoxically, the activity of the Wnt reporter was impaired in the CM of *Maz* mutants. One possible explanation for this discrepancy is a biphasic mode of regulation in which an increase of *Sfrp2* at the border of the CM initially favors Wnt/β-catenin signaling by bringing ligand and receptor together, but once Sfrp2 reaches a specific threshold it inhibits this activity. This idea is supported by the observation that forced expression of *Sfrp1* in the wing imaginal disc of *Drosophila* impairs spreading of the *Wingless* (*Drosophila Wnt* homolog) gradient. This impairment results in an inhibition of expression of *Wingless* target genes that require a high level of *Wingless* and an activation of expression of those genes that require low levels of *Wingless* (Esteve et al., 2011).

Alternatively, the variations in the CM phenotype (including both expansion and reduction of the CM) might be explained by Maz regulation of other unidentified spatio-temporal factors regulating Wnt signaling to control multiple aspects of peripheral eye differentiation. For example, tsukushi (TSK; also known as TSKU), a group of soluble molecules belonging to the small leucine-rich proteoglycan (SLRP) family, inhibit Wnt2b activity and repress the induction of peripheral eye character by quenching Fzd4 activation, thereby regulating the size of peripheral structures and especially the CB (Ohta et al., 2011). Another possibility is that *Maz*, which is also expressed in the periocular surface ectoderm and lens epithelium, might affect the Wnt signaling from the surface ectoderm to the retina that promotes the morphogenesis of the CM (Carpenter et al., 2015).

Reciprocal interactions to establish eye tissue boundaries occur *in vitro*, showing a remarkable plasticity between neuroretina-RPE fates during eye development (Kuwahara et al., 2015; Rowan et al., 2004). In optic cups derived from mouse embryonic stems cells, aggregate interactions between the neuroretina and RPE promote self-organization of the CM. This self-organization is induced by Wnt agonists and is reversed by Wnt inhibitors. However, some heterogeneity in the responsiveness to the reversal of this trigger was also observed. In some aggregates, RPE and the neuroretina were co-generated and CM was self-organized between them. In some, the aggregates became entirely neuroretina, and yet others were resistant to the reversal trigger and remained as RPE. These studies suggested that neuroretina-RPE self-organization follows the ‘bi-stability’ mechanism in the regulatory network (Sasai, 2013). This occurs when state A and state B inhibit each other, whereas each state activates itself. Under these circumstances, the system tends to give a black or white, but not a gray, outcome. This mechanism produces a sharp boundary of domains similar to a toggle switch. In this context, Maz as a regulator of Wnt activity might have an important role in the specification of the CM by excluding the expression of *Sfrp2* in the CM, which might involve activation, autoregulatory loops and mutual inhibition of Wnt2b and Fzd4 in the peripheral retina*.* Small differences in the expression levels of these key genes are likely to have a pivotal role in the specification of either the neuroretina or CM in the periphery of retinal epithelium.

Additionally, the expansion of the proximal *Msx1* expression domain in the CM observed in some of the *Maz* mutants suggested that the spatial patterning of the mouse CM in the distal and proximal domains is also altered (Belanger et al., 2017; Kuwahara et al., 2015; Marcucci et al., 2016). It is therefore possible that Maz also regulates the proximodistal patterning of CM. As *Msx* genes are targets of Wnt signals (Willert et al., 2002), and β-catenin induces expression of both *Msx1* and *Otx1*, it is possible that this also takes place through the regulation of the canonical Wnt cascade.

**Fig. 7.**
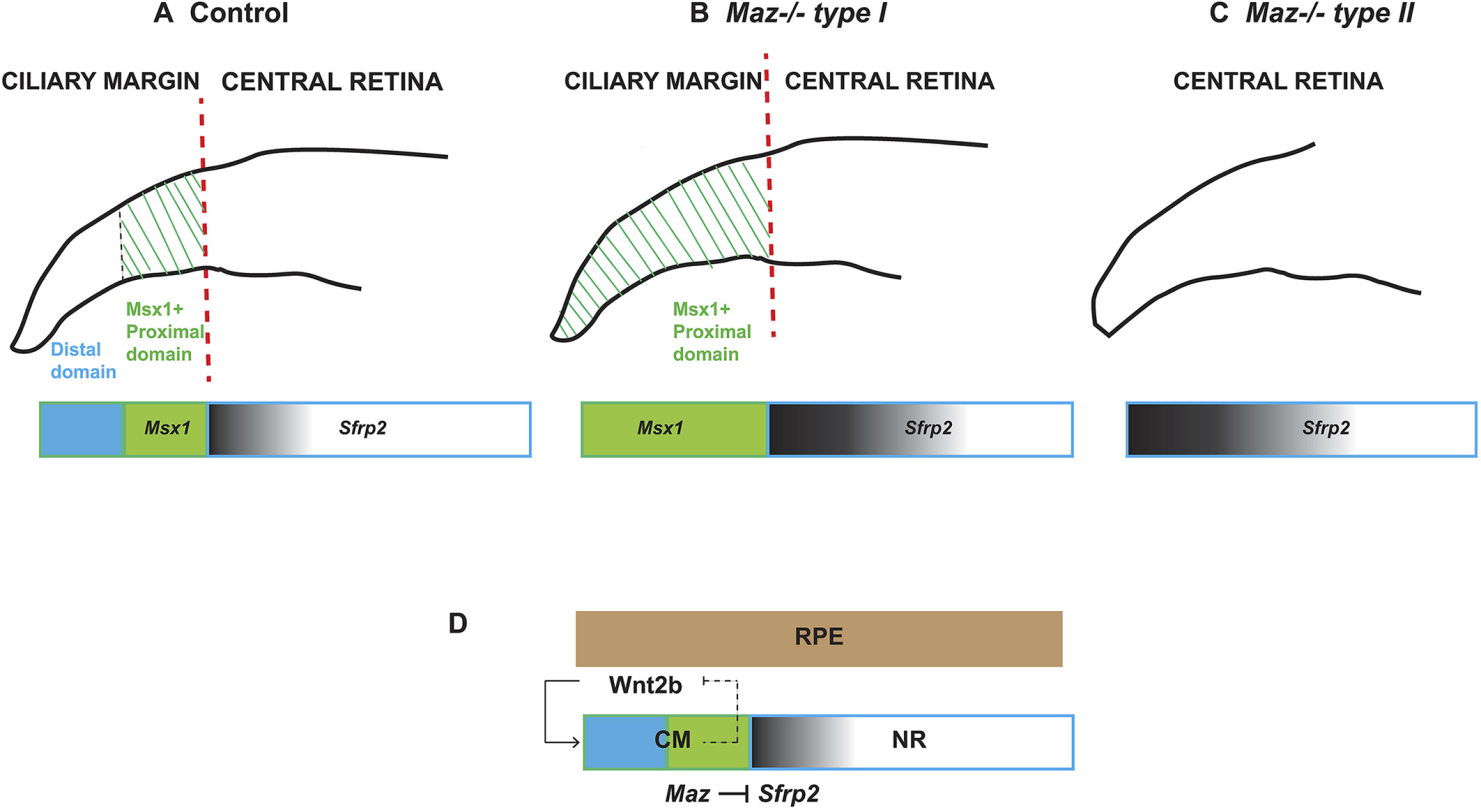
**A model of the hypothetical relationship of mutual** **interactions between CM and the neuroretina.** (A-C) Schematic representation of domains in the CM and the central (neural) retina sharp boundary in control retinas (A) compared with the two different types of *Maz*^−/−^ mutant retinas (B,C). Molecular markers represented as boxes: *Msx1* (green box) expression in the control retina is confined to the distal domain of the CM. *Sfrp2* (blue box with gray gradient) representing the high-low gradient in the central retina and the changes observed in *Maz* mutant type I (B) and type II (C). (D) Schematic representation of the proposed role of *Maz* as a regulator of *Sfrp2* expression (a diffusible Wnt agonist/antagonist) in the CM. The CM and neuroretina (NR) inhibit each other's development through Wnt signaling (Wnt2 and Fzd4) and Sfrp2, which promotes CM at the cost of NR differentiation. CM reinforces its own fate by augmenting the expression of *Msx1* and *Otx1*, whereas *Maz* keeps the expression of *Sfrp2* to the central retina. The activation of this pattern requires auto-activation and its inhibitors for negative feedback.

On the basis of our observations, we propose that Maz has an important role as a regulator of Wnt activity in the promotion of CM fate. In this working model (Fig. 7), *Maz* regulates Wnt/β-catenin activity in the CM by limiting expression of *Sfrp2* to the central retina. Modulation of *Sfrp2* expression in the CM might be crucial, not only for the determination of cell fate in the border between peripheral and central retina, but also for the correct proximodistal patterning of the CM. However, the involvement of other Maz mechanisms in the context of eye development, in addition to the canonical Wnt signal, cannot be ruled out. Indeed, many studies of Maz in other settings have illustrated other potential roles, such as competing or interacting with other transcription factors (Sohl et al., 2010; Morii et al., 2002; Lee et al., 2016). Of particular note in the eye, developmental vascular regression is regulated by Wnt/β-catenin and MYC-CDKN1A (Nayak et al., 2018; Rao et al., 2013). Interestingly, *Myc* is transcriptionally repressed by Maz, suggesting that it might have additional roles in the regulation of the Wnt cascade (Izzo et al., 1999).

### *MAZ* is one of the primary dose-sensitive genes associated with chromosome 16p11.2 ocular anomalies in humans

On the basis of our studies showing a gene-dose-dependent regulatory role of *Maz* in mouse eye morphogenesis, we evaluated *MAZ* as a candidate gene for human eye disease. Several cases of CNVs associated with anophthalmia/microphthalmia were reported in the literature in the 16p11.2 chromosomal region, where *MAZ* is located (Bardakjian et al., 2010; Bardakjian and Schneider, 2011; Hernando et al., 2002). We found that heterozygous CNV deletions and duplications of chromosomal region 16p11.2 are associated with ophthalmic comorbidities, including microphthalmia, coloboma, cataracts and others, in nearly 6% of symptomatic individuals carrying 16p11.2 CNVs in DECIPHER. These results are consistent with previously reported associations in the literature (D'Angelo et al., 2016; Zufferey et al., 2012).

Although the 16p11.2 CNV locus contains more than 25 genes and a simple phenotype-genotype correlation could not be inferred, we identified seven genetic *MAZ* variants present in patients with variable eye anomalies. Two of these, R276L and A404V, are predicted to produce deleterious structural and functional modifications of the MAZ protein. Given that non-penetrance has been observed for the variant P76Q in the mother of the affected individual, it is possible that this individual is non-penetrant or the phenotype is milder. The non-penetrance of such a variant is similar to the incomplete penetrance observed in *Maz* mutants and might reflect effects of the background or compensation by paralogs. Similar non-penetrance has been observed in families with pathogenic variants of *FZD5*, a gene in the WNT pathway that causes coloboma (Liu et al., 2016). However, additional functional studies are necessary to understand the significance of these variants.

In summary, our study highlights *MAZ* as an important dose-sensitive gene responsible for abnormal ocular development in 16p11.2 syndrome. This is supported by an *in vivo* loss-of-function mouse model, showing that *Maz* is required for eye morphogenesis. The identification of *MAZ/Maz* function on eye development in both human and mouse broadens opportunities to elucidate the disease mechanisms and treatment for ocular malformations.

## MATERIALS AND METHODS

### Animals

All animal protocols were reviewed and approved by the Institutional Animal Care and Use Committee (IACUC) at Baylor College of Medicine, and the experiments were performed in adherence to the National Institutes of Health Guidelines on the Use of Laboratory Animals. Generation of *Maz* knockout mice (*Maz*^−/−^) was previously described (Haller et al., 2018) and produced using the CRISPR-Cas9 methodology. Two single-guide RNAs flanking *Maz* exon 2 to 4 were used. Cas9 recombination resulted in a deletion of the zinc-finger C2H2 domains, producing a truncated protein unable to bind DNA. Animals were maintained in a C57BL/6J background. *Maz*^+/−^ crosses were used to generate *Maz*^−/−^ embryos, as the deletion of *Maz* resulted in perinatal lethality.

TCF-Lef1-LacZ reporter mice were obtained from The Jackson Laboratory. Heterozygous TCF-Lef1-LacZ mice with a CD1 background were crossed back two generations to *Maz*^+/−^ mice to generate *Maz*^+/−^/TCF-Lef1-LacZ mice with a C57BL/6J and CD1 mixed background. Heterozygous *Maz*^+/−^/TCF-Lef1-LacZ mice were crossed to obtain *Maz*^−/−^/TCF-Lef1-LacZ mice and control *Maz*^+/+^/TCF-Lef1-LacZ mice, respectively.

### Immunohistochemistry

Embryos were fixed in 10% formalin, dehydrated in a progressive graded series of ethanol and embedded in paraffin. Sectioned heads were deparaffinized, rehydrated in PBS and treated with antigen retrieval solution (10 mM sodium citrate, pH 6) in a microwave for 20 min. After a rinse with PBS, sections were circled with a PAP pen and incubated with blocking buffer (5% bovine serum albumin/0.5% Tween-20 in PBS) for 1 h at room temperature in a humidifier chamber. Next, sections were incubated overnight with one of two antibodies: phosphohistone H3 (1:500, Upstate) or Pax6 (1:200, DSHB). The next day, slides were incubated with the corresponding rabbit Alexa Fluor 594 or mouse Alexa Fluor 488 secondary antibodies for 1 h and followed by three washes. Sections were mounted with anti-fade mounting medium and visualized by fluorescence microscopy.

### Proliferation and cell death

Incorporation of EdU was analyzed in six embryos by genotype and each eye analyzed separately (*n*=12), as previously reported (Mead and Lefebvre, 2014) with some modifications. The Click-iT^®^ EdU Alexa Fluor^®^ 488 Imaging Kit (Invitrogen) was used, which contains all components needed to label DNA-synthesizing cells and to detect EdU incorporated into DNA. A pregnant mouse was injected intraperitoneally with 10 µl EdU solution/g body weight. After 2 h of EdU labeling, heads of embryos were fixed with 10% buffered formalin for 2 h at room temperature. The heads were then dehydrated and embedded in paraffin. Sections (7-10 µm) were used to perform all the instructed steps of the assay in the dark. For quantification of EdU-positive cells in the CM, cells were counted on three sections through the medial plane of the lens for a total of six animals per genotype.

Cell death was detected using the *In situ* Cell Death Detection Fluorescein Kit (Sigma-Aldrich) in four embryos. Manufacturer's instructions were followed for the labeling reaction on eye sections. After permeabilization and labeling with TUNEL reaction mixture, sections were analyzed by fluorescence microscopy.

### Whole-mount ISH

Mouse embryos (E9.5-E12.5) were dissected and fixed overnight with 4% paraformaldehyde. Embryos were then dehydrated and re-hydrated in methanol series and treated with 10 µg/mL of proteinase K for 10 min. ISH was performed overnight with digoxigenin (DIG) antisense or sense probes. Post-hybridization washes and DIG antibody incubation were performed according to a standard protocol (Wilkinson, 1992). Signal was visualized with BM purple (Roche).

### ISH to sections

Embryos (E9.5-E16.5) were fixed in 4% paraformaldehyde phosphate buffer overnight. Tissue was rinsed in PBS and embedded directly in Optical Cutting Temperature (OCT) compound (VWR Chemicals) or progressively dehydrated in ethanol and embedded in paraffin. Paraffin sections from embryos (E14.5-E18.5) were hybridized overnight with RNA DIG-labeled probes at 65°C. The next day, sections were incubated in 5× SSC with RNase (1 µg/mL) at 37°C, covered with blocking buffer (BMB, Roche) and incubated with alkaline-phosphatase-coupled DIG antibody (1:5000) overnight. Sections were washed and signal was visualized with BM purple (Roche). Cryostat sections were processed for ISH using a standard protocol (Blackshaw, 2013).

### RNA extraction and qPCR

RNA from E16.5 eyes was extracted using Qiagen RNeasy mini kit. Complementary DNA (cDNA) was obtained using High-Capacity RNA to cDNA Kit (Applied Biosystems). qPCR reactions were performed in 96-well plates with the SYBR Green PCR Master Mix. Reactions were performed in triplicate, using three independent samples. Samples were normalized to glyceraldehyde-3-phosphate dehydrogenase (GAPDH). Relative expression software tool (REST) analysis (Pfaffl et al., 2002) was performed to determine level of significance.

### X-gal staining

To detect X-gal staining, embryonic heads (E12.5-E16.5) or eyes (E17.5) were fixed in a 4% buffered paraformaldehyde (PFA) solution for 10 min on ice and rinsed twice in PBS for 5 min. Fixed samples were incubated in a staining solution (5 mM potassium ferricyanide, 5 mM potassium ferrocyanide, 2 mM MgCl_2_, 0.01% sodium deoxycholate and 0.02% nonident P-40 plus 1 mg/mL X-gal in PBS) overnight. The next morning, samples were rinsed in PBS and post-fixed in 4% PFA before paraffin embedding and sectioning.

### CNV overlap mapping

DECIPHER database (https://decipher.sanger.ac.uk/) was searched to identify patients with 16p11.2 CNVs harboring eye phenotypes.

### ES

For the ES cohort, data from ∼11,500 individuals undergoing clinical ES at Baylor Genetics Laboratory were examined for all called variants in *MAZ* (Yang et al., 2014).

### Statistical analysis

Results comparing two groups were analyzed either by Student's *t*-test or by Mann–Whitney *U-*test. *P*-values are indicated in the figure legends.

## Supplementary Material

Supplementary information
