# Supplementary material for: The transcription factor Maz is essential for normal eye development
Source: Dis Model Mech. 2020 Aug 18;13(8):dmm044412. doi: 10.1242/dmm.044412 (PMC7449797; doi:10.1242/dmm.044412)
Supplement: Supplementary information [file dmm-13-044412-s1.pdf]

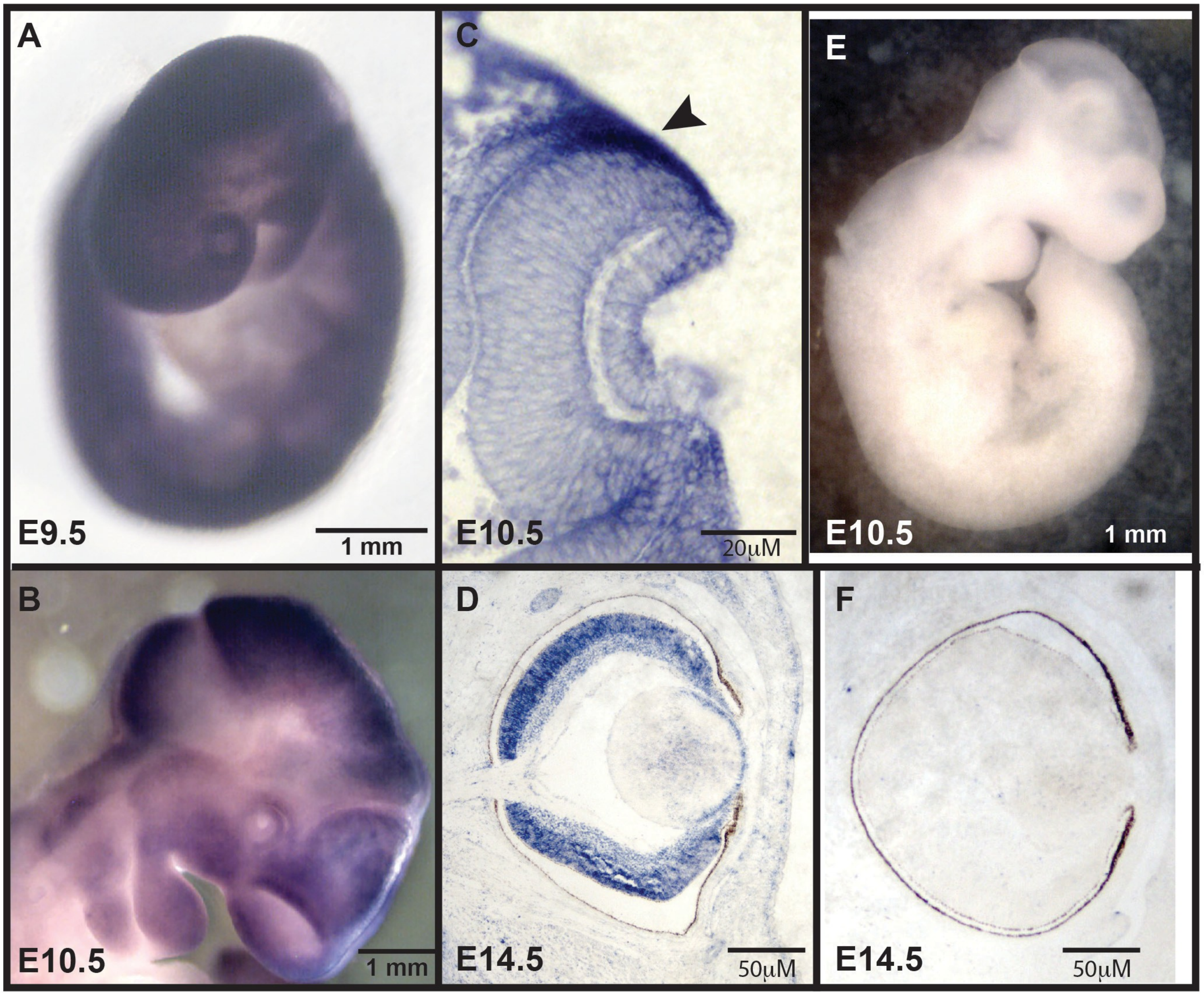

Fig. S1. *Maz* expression during mouse development. Whole mount ISH (6 eyes per age) of *Maz* antisense probe to E9.5 (**A**) and E10.5 (**B**). Section ISH of mouse developing eyes from E10.5 (**C**), E14.5 (**D**). At E9.5, *Maz* is expressed widely in the entire embryo and robustly expressed in the eye region. At E10.5, strongest *Maz* expression is observed in the dorsal portion of the optic cup (arrow) (**C**). At E14.5, *Maz* is expressed in the retina and the anterior lens epithelium (**D**). As a control, *Maz* sense probe was hybridized to E10.5 embryo and no signal was detected (**E**). The antisense *Maz* probe was hybridized to a *Maz* mutant E14.5 eye and no signal was detected (**F**).

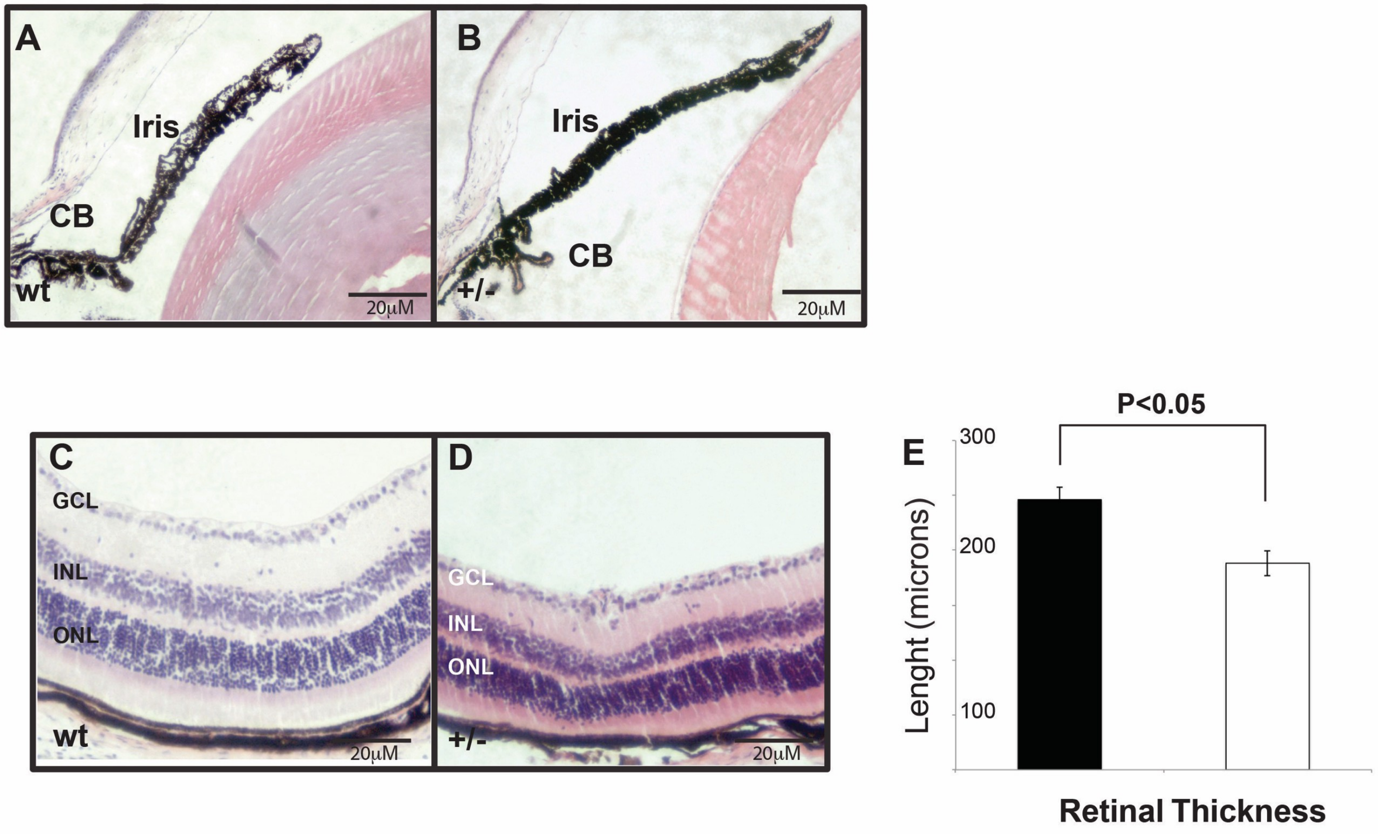

Fig. S2. *Maz* haploinsufficiency causes an overall reduced thickness of the retina and closed anterior chamber angle in adult mice. In the adult *Maz* heterozygous eye (B) the anterior chamber angle is closed when compared to the open angle in the wild type eye (A). Sagittal sections (n= 6-8 eyes per genotype) of adult wt (C) and *Maz*$^{+/-}$ retinas (D) showing the overall reduction in thickness of *Maz*$^{+/-}$ retina compared to wt. Retinal thickness was significantly decreased in *Maz*$^{+/-}$ (E). Error bars are s.e.m. of n = 7-8 eyes per group. *P<0.05, (Mann-Whitney U test). CB, ciliary body; ONL, outer nuclear layer; INL, inner nuclear layer; GCL, ganglion cells layer.

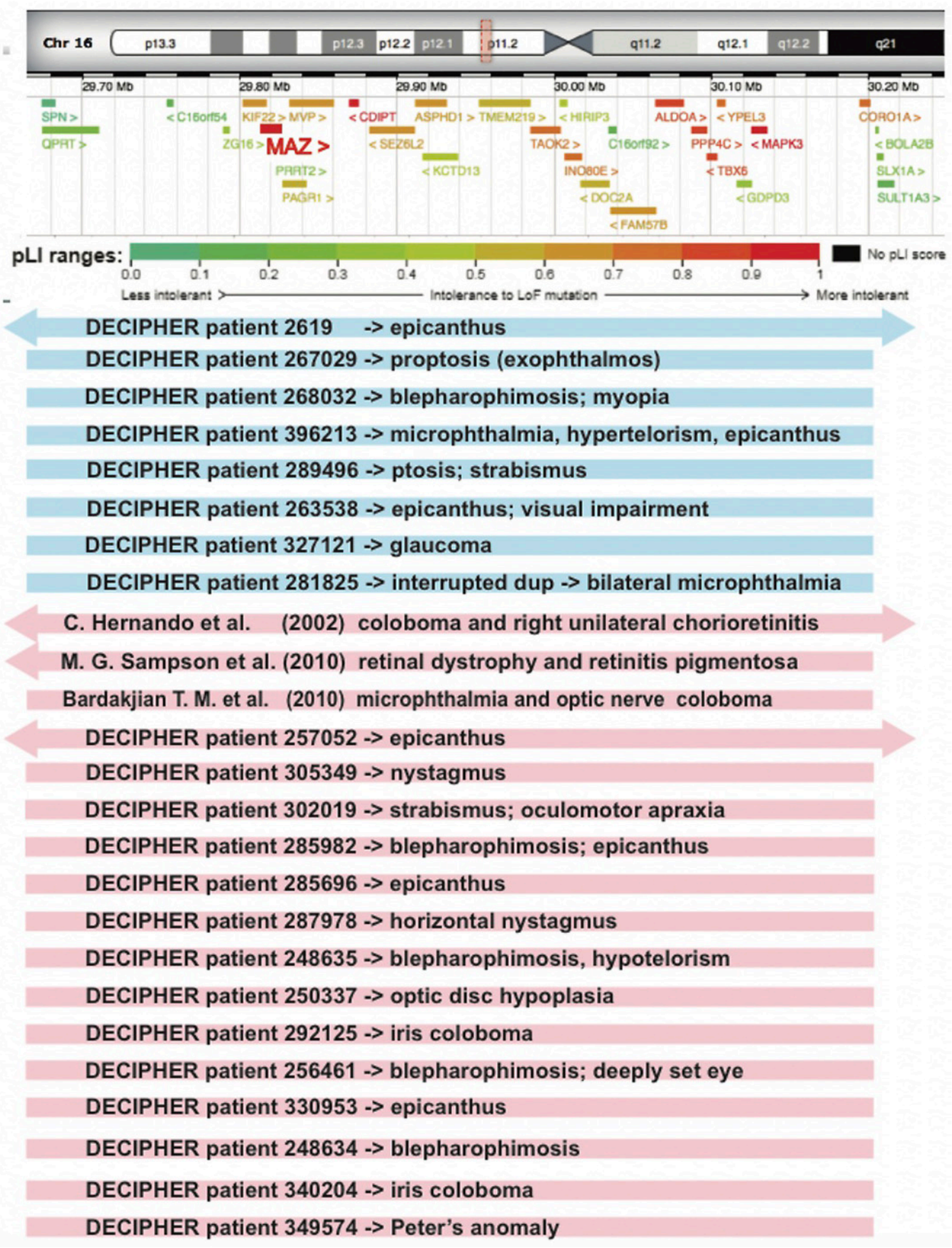

Fig. S3. Microdeletions and duplications at chromosome 16p11.2 in patients with abnormal eye phenotype that were identified in literature or in the DECIPHER database. Graphical representation of the chromosome 16 with a red dashed box demarcating the region associated with autism and cognitive delays and more recently with eye abnormalities. The 16p11.2 core region includes 25 protein-coding genes represented in their relative chromosomal positions. Genes are color coded by their haploinsufficiency rating according the DECIPHER database. The blue boxes correspond to patients carrying microduplications while red boxes represent microdeletions. Arrows indicate that some copy number variations extend beyond the region represented in the map.

GENOTYPE	NORMAL	LEFT EYE	R IGHT EYE	BILATERAL	ANOPHTHALMIC	TOTAL
WT	104	0	4	2	0	110
			abnormal eye shape (3)<br>coloboma (1)	microphthalmia (1)<br>D-V rotated eye (1)		
Maz +/-	133	4	18	13	0	168
		D-V rotated eye (4)	microphthalmia (4)<br>coloboma (7)<br>D-V rotated eye (5)<br>R microphthalmia/ L D-V rotated (2)	microphthalmia/only R coloboma (1)<br>microphthalmia (3)<br>coloboma (1)<br>D-V rotated eyes (3)<br>R absent/ L coloboma (2)<br>R absent/ L D-V rotated (1)<br>R microphthalmia/ L absent (1)<br>R microphthalmia/ L coloboma (1)		
Maz -/-	17	4	26	31	12	90
		D-V rotated eye (2)<br>coloboma (1)<br>Dorsal area no RPE (1)	microphthalmia (4)<br>coloboma and microphthalmia (2)<br>coloboma (3)<br>D-V rotated eye (17)	R/L microphthalmia (12)<br>R/L coloboma and microphthalmia (2)<br>R/L coloboma (2)<br>D-V rotated eyes (7)<br>R absent/L coloboma (3)<br>R absent/L microphthalmia (1)<br>R microphthalmia/L coloboma (2)<br>R microphthalmia/both coloboma (1)<br>R coloboma/L D-V rotated (1)		

Table S1. Frequency of the eye phenotypes observed in the heterozygous and homozygous mouse embryos in the C57Bl/6J background.

Disease Models & Mechanisms • Supplementary information

	Total	WT	*Maz* +/-	*Maz* -/-	p-value
F0-254/F1-724	114	37 (0)	56 (11)	21 (17)	< 0.00001
F0-248/F1-729	161	43 (1)	70 (7)	47 (25)	< 0.00001
F0-258/F1-867	95	21 (2)	53 (14)	21 (18)	< 0.00001
F0-283/F1-573	89	29 (1)	39 (10)	21 (16)	< 0.00001

Table S2. Penetrance of the eye phenotype in the four independent C57 BL/6J *Maz* -deficient lines; (n) - number of affected individuals.

Disease Models & Mechanisms • Supplementary information
